# Wharton's Duct Sialolith of Unusual Size: A Case Report with a Review of the Literature

**DOI:** 10.1155/2014/373245

**Published:** 2014-10-27

**Authors:** Nithin Mathew Cherian, Sankar Vinod Vichattu, Ninan Thomas, Aabu Varghese

**Affiliations:** Department of Oral and Maxillofacial Surgery, Mar Baselios Dental College, Kothamangalam, Ernakulam, Kerala 686691, India

## Abstract

There is an increased incidence of submandibular gland duct developing sialoliths. Among them the sialoliths attaining a size of more than 1.5 cms are rare. Here we present a case with an abnormally sized sialolith in Wharton's duct and a review of the literature about the abnormally sized sialoliths and various anatomical and physiological considerations of the duct which contribute to the higher incidence of sialolith in the duct.

## 1. Introduction

Sialolith is one of the most common diseases of salivary glands. It is estimated to have a frequency of 0.15% in the adult population with slight male predilection [1, 2]. Most sialoliths (80–90%) develop in the submandibular gland: 5–10% develop in the parotid gland and the remainder in the sublingual and minor salivary glands [3–5]. Sialoliths are always found in the distal portion of the duct or at the hilum of the submandibular gland with a few in parenchyma [6]. Salivary stasis and salivary viscosity, rather than the calcium content of the individual salivary gland secretion, play a significant role in its development [1]. Commonly, sialolith measures from 1 mm to less than 1 cm. They rarely measure more than 1.5 cm. Giant sialoliths are rare [7]. Literature search found 30 cases, each measuring more than 1.5 cm or more have been published (Table 1). The aim of this paper is to present a case of an unusually sized sialolith and review of the literature on large sialoliths (1.5 cm or larger).

## 2. Case Report

A 36-year-old male patient reported to the department of Oral and Maxillofacial Surgery, Mar Baselios Dental College, Kothamangalam, complaining of pain and swelling in the floor of the mouth for 1 year. He also gave a history of intermittent increase in the swelling in the early morning and pain during eating which later subsides on its own. The pain was of moderate variety that the patient could tolerate. There was no associated history of fever, malaise, or burning sensation in the oral cavity.

**Figure 1:**
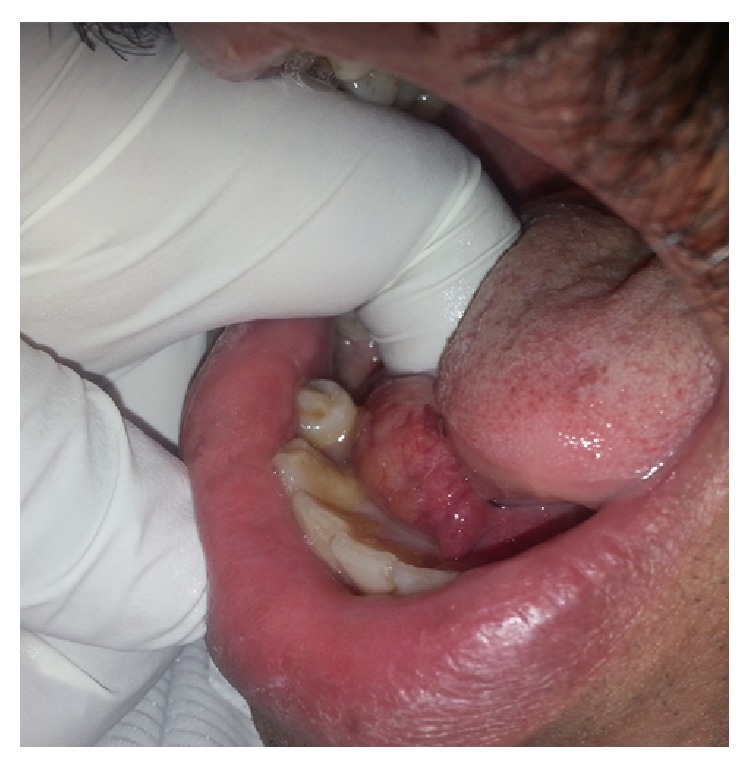
Intraoral palpation.

On extraoral examination no relevant findings were seen. Intraoral examination revealed a swelling of size 3 × 1 cm extending anteroposteriorly and mediolaterally on the right floor of the mouth from lingual frenum to the second premolar region (Figure 1). Overlying mucosa is found to be normal with no salivary obstruction. On palpation the swelling was found to be hard in consistency and nontender. The lesion was not fixed to the underlying structures and it was not pulsatile. No purulent discharge was detected from the duct orifice and the salivary flow was found to be normal.

**Figure 2:**
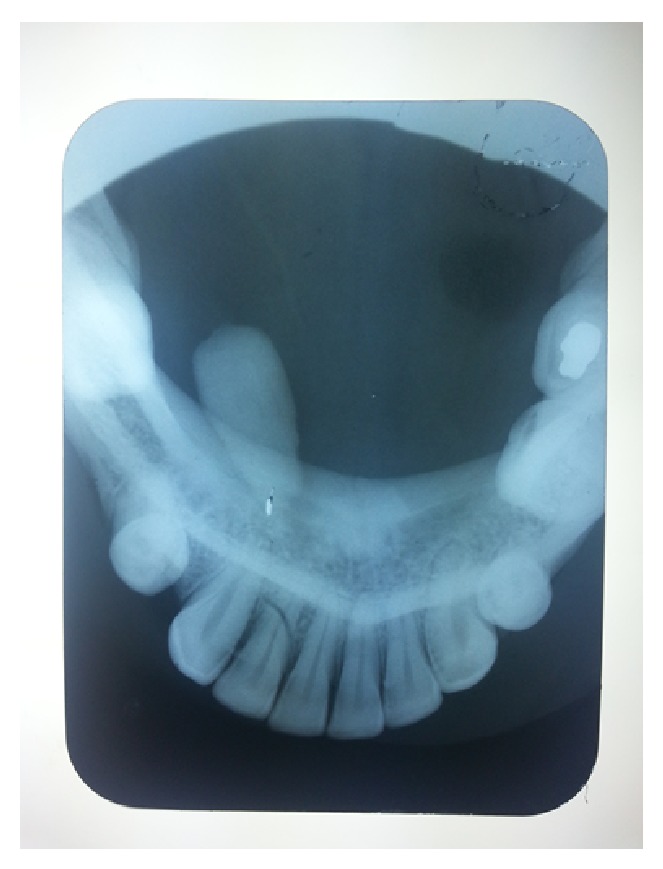
Occlusal radiograph.

Radiographic examination with a panoramic radiograph and occlusal radiograph revealed a radiopaque mass of size 3 × 1 cm extending anteroposteriorly and mediolaterally from the mandibular lateral incisor region to premolar region in the floor of the mouth, suggestive of a sialolith (Figure 2).

**Figure 3:**
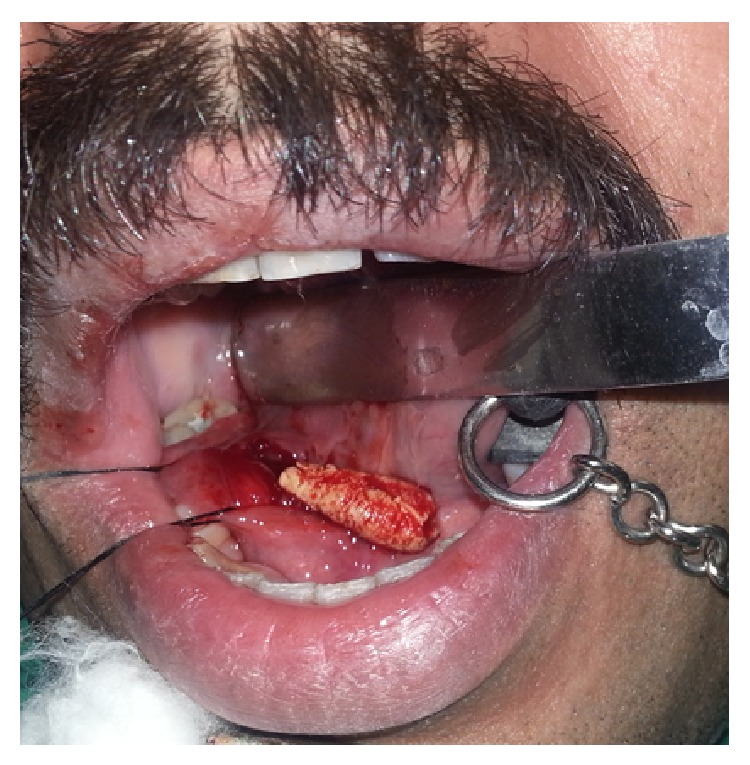
Sialolith evacuated.

**Figure 4:**
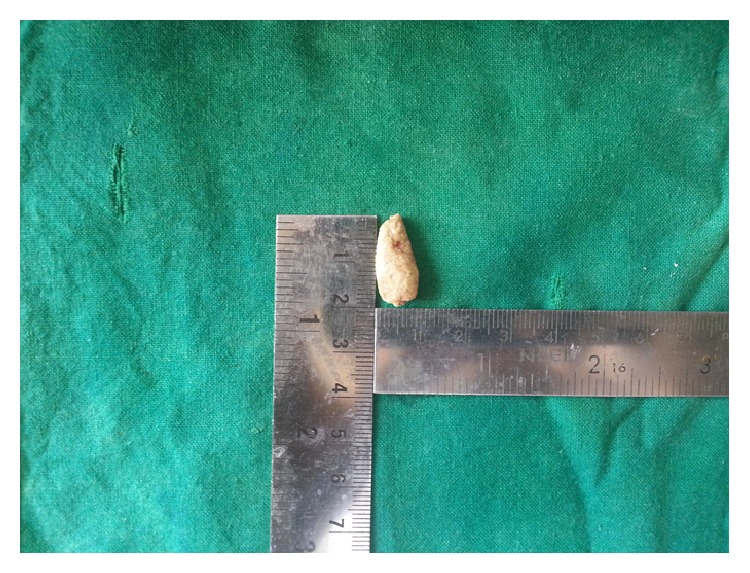
Sialolith measuring 20 mm.

After induction of local anesthesia, retraction suture was placed around the duct distal to the stone, which was then retracted anteriorly. A mucosal incision was placed and careful blunt dissection of the tissues was done and sialolith was located. A longitudinal incision through the superior duct wall overlying the sialolith was placed and the sialolith was evacuated (Figures 3 and 4). Saline irrigation and milking of the gland was done to remove any small residual stones or mucin plugs. Approximation of the wound was done with a few 3–0 vicryl sutures. Following the postoperative instructions the patient was recalled after seven days for review. The healing was found to be satisfactory and salivary flow was found to be normal and patient was relieved of the symptoms.

## 3. Discussion

Sialolithiasis is a rare disease with male predilection. The disease can occur at any age, but it appears more frequently in the third to sixth decades of life. Submandibular glands are more commonly affected than parotids with the duct as a more common site for occurrence of sialolith [7].

According to Harrison et al. the formation of the nucleus of sialolith in the submandibular glands is secondary to sialadenitis and is related to the duration of symptoms of sialadenitis [8]. According to them during chronic submandibular sialadenitis inflammatory swellings would lead to the partial obstruction of a large duct with stagnation of secretory material rich in calcium. This would form a calcified core and later when this grows, it would become a sialolith.

**Figure 5:**
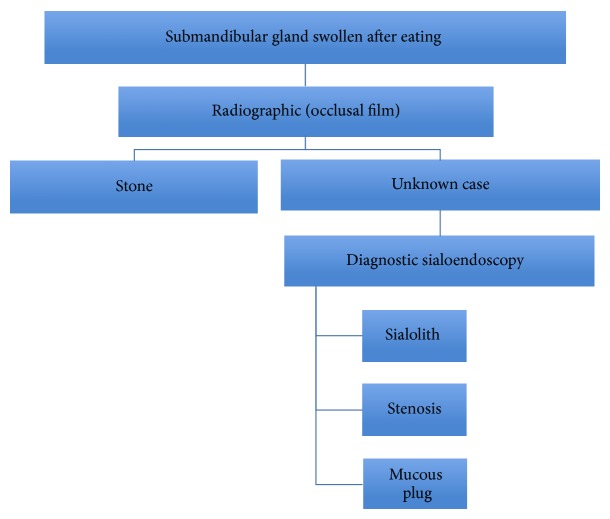
Diagnostic algorithm [16].

Figure 5 shows signs and symptoms which include swelling, anatomical asymmetry, size fluctuation, usually rapid onset and partial resolution over one to several hours, residual glandular swelling, decreased salivary flow as compared to the contralateral gland, pain which intensifies during meal times or when salivary flow is stimulated, swelling and erythema of submandibular papilla for distal stones, and unusually suppuration or localized cellulitis [9].

Radiographically it can be seen as a radiopaque structure, which may be homogenous, or with a laminated structure. Some may be radiolucent also [9]. As seen in Table 1 every one of them occurred in male patients; with the exception of one case all large sized sialoliths were located in the submandibular duct (94.4%) and only an isolated case was found with in Stensen duct of parotid salivary gland.

Several factors seem to be involved in the higher incidence in the submandibular gland compared to the parotid.

1. The submandibular duct is wider in diameter and longer than the Stensen duct.
2. The salivary flow in the submandibular gland is against gravity.
3. The submandibular salivary secretion is more alkaline compared with pH of the parotid saliva.
4. The submandibular saliva contains a higher quantity of mucin proteins whereas parotid saliva is entirely serous.
5. Calcium and phosphate content in submandibular saliva are higher than in other glands.

Generally sialoliths are thought to start from retention of saliva in the salivary duct. Latest studies with sialoendoscopy revealed more chances of saliva retention in submandibular duct. The lining of the duct seen endoscopically is white and avascular, and the duct could itself cause partial obstruction.

During sialoendoscopy some special features were found in the lumen and wall of the duct by Yu et al. [5]. One special structure is a sphincter-like mechanism or muscle-like structure [10, 11]. This has a valve-like function and can prevent the foreign body from entering the duct, which is located on the anterior side of submandibular duct, which can be related to the formation of sialolith in the submandibular gland.

Marchal et al. [6, 12, 13] reported the results of examination of 120 submandibular glands and the sphincter was located in the first 3 mm of Wharton's duct. Another special structure is a basin-like structure in the submandibular gland, which expands into the region of hilus on sialoendoscopy. It is also called pelvis-like or coma area. It may slow down the flow of saliva and cause the sediment of inorganic substance to sink and induce gradual formation of a sialolith if a nidus such as a mucus plug or a foreign body exists.

**Figure 6:**
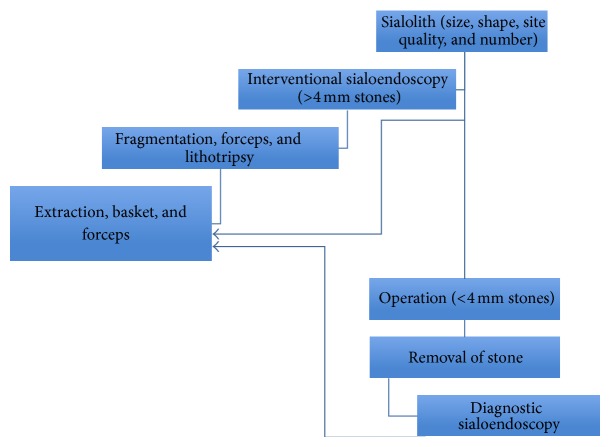
Therapeutic algorithm [16].

Figure 6 shows treatment options for patients with salivary stones which include removal through the oral cavity, interventional sialoendoscopy, and resection of the gland. Treatment choice depends on the site, size, shape, number, and quality of the stones.

### 3.1. Surgical Removal of Salivary Stones from the Submandibular Duct [14, 15]

#### 3.1.1. Intraoral Removal

*Procedure.* The aim of this procedure is to dissect the Wharton's duct, isolate it, and subsequently remove the stone. Rather than blind searching of mobile soft tissue for the stone immobilization of that portion of the oral floor and duct to be exposed is done confining the limits of the stone antero posteriorly. This is done by placement of two deep sutures, one anterior and one posterior to the calcification. There is an option for making the suture radio opaque by impregnating the suture with iodinized oil for view in the occlusal radiograph so that the sutures can be accurately placed.

There is an anatomical feature that can be used for circumscribing the duct. The plica sublingualis, which is an elevated crest of the mucous membrane, is caused by the oral projection of the sublingual gland and is located in the floor of the mouth along the lateral border of the tongue posterior to the lingual frenum. It is closely approximated with anteroposterior course of the duct and is located in a plane directly above or slightly lateral to the pathway of Wharton's duct.

The second anatomical feature is the course of the duct which progressively ascends as it courses anteriorly from the gland to its orifice; the depth to which the suture must be placed can be readily reckoned.

After the placement of the anterior suture the posterior suture is tied tightly to prevent the slipping of the stone posteriorly. The sutures should be of sufficient length so that it is possible to grasp them manually, thus raising the operative site and making it taut. Additional extraoral pressure under the floor of the mouth helps in elevation of the area to be surgically explored. The suture combined with the extraoral pressure will facilitate more accurate and simple dissection of the area.

The cardinal rule in the removal of Wharton's duct sialolith is that because the sialolith is located intraductal it can never be lost if the duct is first located and sufficiently isolated [9]. Direct cutdown on the stones in the longitudinal portion of the duct is ill advised as it can lead to the maceration of the duct making sialodochoplasty impossible and may result in salivary leakage or stenosis [9, 15, 16].

There are also possibilities of the portions of sialolith being lost into the surrounding tissues, resulting in infection. The situations where the cut-down procedure is not only acceptable but also recommended are

1. when the sialolith is present at the ductal orifice—in this situation an incision over the stone will aid in the extirpation of the stone and at the same time will allow for a sialodochoplasty, which is done by suturing the exposed duct walls to their respective adjacent mucosa after the insertion of a lacrimal probe into the duct lumen;
2. when there is a large stone in the submandibular gland, pushing the gland upward and anteriorly, resulting in the projection of the stone prominence intraorally. Incision through the overlying mucosa will result in the projection of the stone prominence intraorally. Incision through the mucosa will result in the exit of the stone because the gland is probably grossly fibrotic and nonfunctioning and no other treatment is likely to be necessary.

After identifying and isolating Wharton's duct and examination of significant anatomic structure an initial incision is made anteriorly in the suture confined area. As the duct ascends anteriorly the movement of the sialolith will be in an anterosuperior direction so that the anterior third is relatively close to the surface mucosa. The duct adjacent to the medial surface of sublingual gland whose superior projection is manifested by the raised plica-sublingualis. A 2 cm incision is made medial and parallel to the plica extending from the cuspid to the second bicuspid region. If made laterally the dissection to locate the duct would perforate and injure the sublingual gland increasing the risk of an iatrogenically induced oral ranula attention needs to be given medial to the second molar in the midportion of the duct to the crossing lingual nerve. A preincision insertion of a lacrimal probe into the duct or careful blunt dissection of the tissues with a curved mosquito hemostat will be successful. Carry the dissection with only slight deviations medially or laterally. Retraction sutures can be placed through the lateral aspect of the incised mucosal tissues and tied to the adjacent teeth. For posteriorly located stones the mucosal incision is extended posteriorly and the duct exposed until a bulge is observed. Follow the duct posteriorly and identify and protect the lingual nerve as it crosses under Wharton's duct. Placing a curved hemostat inferior to it isolates the stone. A longitudinal incision through the superior duct wall overlying the sialolith will result in its evacuation. The patency of the duct is checked by inserting a good sized lacrimal probe, which is then followed with saline intraductal irrigation and milking of the involved gland to remove any small residual stone fragments or mucus plugs.

The completion of the procedure can be done by either a primary closure or sialodochoplasty. If primary closure is done, do not suture the incised duct wall, because this will increase the risk of stenosis. To reduce the extent of oral floor swelling from the salivary leakage and postsurgical edema, a tight mucosal closure is contraindicated and surgical drains are mandatory. A definitive risk for this procedure is increasing the severity of precondition of salivary stasis and also the risk of recurrence. This can be avoided by a dochoplasty. A new fabricated ductal opening is recommended at any location in the horizontal portion of the duct as long as it is posterior to the removed sialolith. The longitudinal superior ductal incision is lengthened posteriorly. The margins are spread laterally, and each side is sutured to their adjacent mucosa with two absorbable fine sutures. If possible a single suture is then placed through the superior wall of the duct at the proximal end of the longitudinal ductal incision to engage the overlying mucosa. Ligation of the duct anterior to the dochoplasty to force salivary flow through the new opening is optional. Periodic duct dilation and sialagogues will ensure a new ductal opening.

#### 3.1.2. Lithotripsy

Electrocorporeal shock wave lithotripsy is an old technique used as a noninvasive technique. Marmary first reported fragmentation of sialolith using shock waves in 1986. Large machines with very broad focus posed a problem at that time, but the development of smaller machines led to finely focused waves, which improved the efficacy of this technique.

Iro et al. used shock wave lithotripsy using piezoelectric lithotripter to treat 35 stones and found that all stones were fragmented but showed only 40% of clearance. Study by Yoshizaki et al. also found only the disintegration of stone into sludge. With the need of advanced armamentarium and poor result this technique does not seem to be effective as a viable routine method of management. Instead of using it as a solo technique adjuvant interventional endoscopy or surgical intervention proved to be effective in the treatment of sialoliths.

#### 3.1.3. Laser Sialolithectomy

Azaz et al. reported sialolithectomy using the Sharplan CO_2_ laser on 47 patients and found the treatment to have excellent results with almost no bleeding, minimal scaring, and little discomfort through the healing period. But there is no added advantage over the conventional surgical management. Being a blind procedure, with the extent of tissue destruction being unknown and the need for specialized equipment with the absence of clear benefit and with the possibility of deleterious effects, this procedure also does not seem to be a feasible technique for removal of sialoliths.

#### 3.1.4. Interventional Sialoendoscopy

The endoscopic system includes diagnostic and interventional sialoendoscopy, a papillary dilator, forceps, grasping wire basket (3–6 wires), and an electrohydraulic lithotripter. Local anesthesia is by lingual nerve block and perfusion of 2% lignocaine through the orifice. The endoscope is rinsed intermittently with a solution of 0.9% sodium chloride. This slightly dilates the duct, cleans the view of the endoscopist, and removes pus, debris, and occasional blood.

The device is inserted through the orifice of Wharton's duct or by a mini incision into the orifice or the anterior part of the duct; the papilla is dilated with dilators of increasing diameter. The first procedure is diagnostic and can explore the ductal system thoroughly. When the stone is located interventional endoscopy is required. Small round stones can be removed by wires or forceps. Larger stones should be fragmented and then be removed by wires or forceps. When there is only stenosis balloon dilatation of the duct can be done and if mucin plugs are present they can be removed by forceps or washed out by continuous lavage through the endoscope. Interventional sialoendoscopy and operation can be used jointly to treat multiple stones. Initial treatment results are found to be satisfactory but long-term results are yet to be explored.

#### 3.1.5. Submandibular Gland Removal

Gland removal is indicated only when small stones are present in the vertical portion of the duct from the comma area to the hilus or within the gland itself that are not surgically accessible intraorally and produce obstructive symptoms [8]. With the availability of interventional endoscope even this can be avoided.

## Figures and Tables

**Table 1:** Unusually sized sialoliths reported in literature.

	Study	Number of cases	Age	Gland	Location	Size
1	Meyers, 1942 [17]	1	50	SM	Duct	50
2	Mustard, 1945 [18]	1	42	SM	Duct	56
3	Allen, 1956 [19]	1	49	SM	Duct	35
4	Cavina and Santoli, 1965 [20]	1	59	SM	Duct	70+
5	Cavina and Santoli, 1965 [20]	1	53	SM	Both	60
6	Hoggins, 1968 [21]	1	52	SM	Paren	50
7	Rust and Messerly, 1969 [22]	1	66	P	Duct	51
8	Rust and Messerly, 1969 [22]	1	58	NR	Paren	35
9	Raksin et al., 1975 [23]	1	52	SM	Duct	55
10	Isacsson and Persson, 1982 [24]	1	48	SM	Duct	36
11	Tinsely, 1989 [25]	1	48	SM	Paren	50
12	Hubar et al., 1990 [26]	1	65	SM	Duct	52
13	Akin and Esmer, 1991 [27]	1	45	SM	Paren	45
14	Paul and Chauhan, 1995 [28]	1	45	SM	Duct	46
15	Bodner, 2002 [29]	1	50	SM	Duct	50
16	Ledesma-Montes et al., 2007 [7]	1	34	SM	Duct	36
17	Gonçalves et al., 2002 [30]	1	52	SM	Duct	22
18	Rai and Burman, 2009 [31]	1	60	SM	Duct	72
19	Miyashita et al., 2013 [32]	1	58	P	Duct	15
20	Yu et al., 2013 [5]	9	15–78	SM and P	Duct	16–29
21	Huang et al., 2009 [9]	1	57	SM	Duct	40
22	This case	1	36	SM	Duct	20

SM, submandibular gland; P, parotid gland; Paren, parenchymal; NR, not reported.
